# Purity of transferred CD8^+^ T cells is crucial for safety and efficacy of combinatorial tumor immunotherapy in the absence of SHP-1

**DOI:** 10.1038/icb.2016.45

**Published:** 2016-07-19

**Authors:** H Angharad Watson, Garry Dolton, Julia Ohme, Kristin Ladell, Miriam Vigar, Sophie Wehenkel, James Hindley, Rebar N Mohammed, Kelly Miners, Rhys A Luckwell, David A Price, R James Matthews, Ann Ager

**Affiliations:** 1 Division of Infection and Immunity, School of Medicine and Systems Immunity University Research Institute, Cardiff University, Cardiff, UK; 2 Vaccine Research Center, National Institute of Allergy and Infectious Diseases, National Institutes of Health, Bethesda, MD, USA

## Abstract

Adoptive transfer of tumor-specific cytotoxic T cells is a promising advance in cancer therapy. Similarly, checkpoint inhibition has shown striking clinical results in some patients. Here we combine adoptive cell transfer with ablation of the checkpoint protein Src homology 2-domain-containing phosphatase 1 (SHP-1, *Ptpn6*). Naturally occurring motheaten mice lack SHP-1 and do not survive weaning due to extensive immunopathology. To circumvent this limitation, we created a novel SHP-1^null^ mouse that is viable up to 12 weeks of age by knocking out IL1r1. Using this model, we demonstrate that the absence of SHP-1 augments the ability of adoptively transferred CD8^+^ T cells to control tumor growth. This therapeutic effect was only observed *in situations* where T-cell numbers were limited, analogous to clinical settings. However, adoptive transfer of non-CD8^+^ SHP-1^null^ hematopoietic cells resulted in lethal motheaten-like pathology, indicating that systemic inhibition of SHP-1 could have serious adverse effects. Despite this caveat, our findings support the development of SHP-1 inhibition strategies in human T cells to complement adoptive transfer therapies in the clinic.

The advent of checkpoint inhibitors has proven to be an exciting development in the fight against cancer. Monoclonal antibodies directed against CTLA-4 (ipilimumab) and PD-1 (nivolumab, pembrolizumab) act via the blockade of key molecular interactions that are known to inhibit T-cell expansion, activation and/or cytoxicity.^1, 2^ These biologic agents are not successful in all patients, although combining anti-CTLA-4 and anti-PD-1 leads to improved outcomes compared with either therapy alone.^3^ This synergistic effect suggests that other inhibitory molecules could be targeted similarly, either singly or in combination, to provide additional benefits. Src homology 2-domain-containing phosphatase 1 (SHP-1, *Ptpn6*) is an inhibitory enzyme expressed at high levels in all hematopoietic cell lineages and at all stages of maturity.^4, 5^ In T cells, SHP-1 regulates interactions with antigen-presenting cells where we have demonstrated its involvement in setting activation thresholds during thymic selection and reducing the requirement for CD28 costimulation.^6, 7^

SHP-1 expression in tumors has been the subject of much investigation. A number of currently available cancer drugs may kill tumor cells by activating SHP-1, leading to inhibition of STAT3 and cell death by apoptosis.^8^ Conversely, immunotherapies have focused on ablation of SHP-1 to counter its inhibitory action on cytotoxic T cells.^9^ The clinically available active-site SHP-1 inhibitor sodium stibogluconate, which also targets the related phosphatase SHP-2, is currently being investigated as a potential cancer therapeutic.^10^ A number of other small molecule protein tyrosine phosphatase inhibitors are being investigated as cancer therapeutics. However, none of them are specific for SHP-1,^10, 11, 12, 13, 14^ and the systemic administration of a multiple phosphatase inhibitor risks off-target effects. Indeed, we show here that the absence of SHP-1 in non-CD8^+^ hematopoietic cells is responsible for fatal lung pathology. Targeting a specific cell type through genetic manipulation reduces such risks to patient safety. Here we demonstrate the improved ability of SHP-1-deficient T cells to limit tumor colonization of the lungs and control the growth of solid tumors in comparison with T cells expressing SHP-1.

## Results and discussion

### Introduction of IL1r1^−/−^ does not affect the anti-tumor properties of SHP-1^null^ T cells

**Figure 1:**
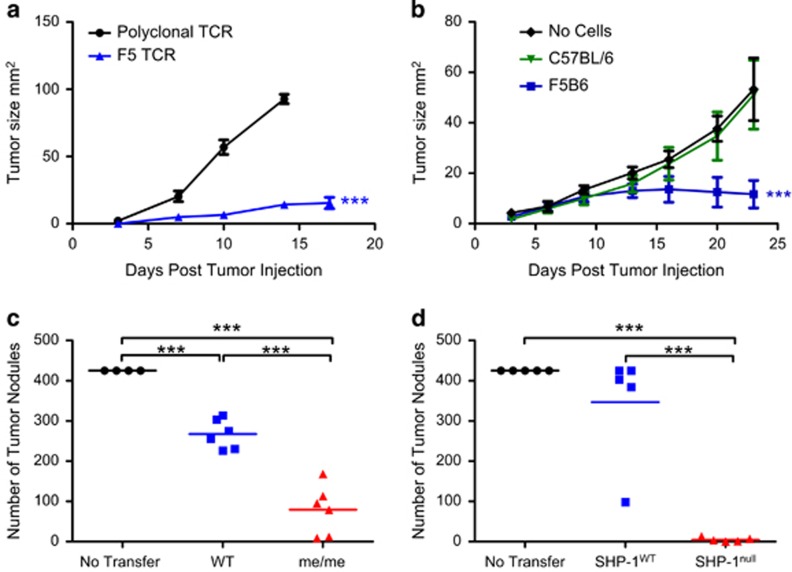
CD8^+^ T cells lacking SHP-1 prevent tumor metastasis formation. (**a**) 5 × 10^5^ B16-NP68 cells were injected subcutaneously into F5B6 hosts or hosts with polyclonal TCRs (C57BL/6) (*n*=3–5). Differences in tumor growth rate were calculated by linear regression. ^***^P⩽0.001. (**b**) C57BL/6 mice received B16-NP68 tumor cells as before. Seven days after tumor injections, mice were sublethally irradiated and given NP68 peptide in IFA with or without 2.25 × 10^5^ naive CD8^+^ T cells (*n*=7). Tumor growth rates were compared using linear regression. ^***^P⩽0.001. (**c**) Tumor nodule enumeration following treatment with F5me/me or F5WT CD8^+^ T cells. One point=one mouse. Significance was calculated using one-way ANOVA with Tukey's *post hoc* test. ^***^P⩽0.001. (**d**) Tumor nodule enumeration following treatment with SHP-1^null^ or SHP-1^WT^ CD8^+^ T cells. Significance was calculated using one-way ANOVA and Tukey's *post hoc* test.

To ensure that SHP-1^null^ and wild type (WT) SHP-1-sufficient cells expressed equivalent levels of an identical T-cell receptor (TCR), we used the transgenic F5 mouse, in which more than 90% of CD8^+^ T cells express a TCR specific for the internal influenza nucleoprotein antigen NP68.^15^ In addition, we engineered a B16F10 murine melanoma cell line to express the NP68 peptide fused with green fluorescent protein (GFP) as well as a luciferase reporter gene. These cells will be referred to as NP68-B16. The B16 tumor line is poorly immunogenic, and mice with polyclonal TCRs were unable to control the growth of subcutaneously grafted NP68-B16 cells, in contrast to F5 mice (Figures 1a and b). Similarly, adoptive transfer of F5 CD8^+^ cells, but not polyclonal CD8^+^ T cells, controlled tumor growth (Figure 1b).

The naturally occurring motheaten (me) mouse is null for SHP-1. However, the absence of SHP-1 across all lineages produces severe pathology, and mice homozygous for the me locus (me/me) die before weaning, mainly as a result of lung pathology.^16^ We employed an assay referred to as a metastatic model, in which tumor cells are introduced intravenously, causing metastasis-like colonization of the lungs without a primary solid tumor. Mice treated prophylactically with me/me CD8^+^ T cells developed significantly fewer metastatic tumor nodules in the lung than mice treated with the corresponding WT cells (Figure 1c). However, limited numbers of me/me T cells are available for transfer in this model due to the severe pathology of me/me mice. There is convincing evidence that the severe cutaneous pathology observed in me/me mice is largely myeloid-driven and reliant on signaling via interleukin 1 (IL1).^17^ An IL1r1 knockout was therefore crossed with the me/me line homozygous for the F5 TCR. The F5^+/+^IL1r1^−/−^me/me mice, hereafter called SHP-1^null^, survived up to 12 weeks of age, exceeding the life expectancy of even the motheaten viable strain.^18^ Skin and lung pathology was still detectable in these animals, but was highly attenuated. However, despite the attenuated pathology, birth rates of the SHP-1^null^ mice were significantly lower than expected (*P*⩽0.005) at only 15 from 130 pups being homozygous motheaten from matings of F5^+/+^ IL1r1^−/−^me/me mice. In addition, ~30% of all pups died before weaning, suggesting developmentally related pathology.

To establish that tumor prophylaxis mediated by me/me CD8^+^ T cells was maintained in the absence of IL1 signaling, the metastasis experiment was repeated using magnetically isolated SHP-1^null^ naive CD8^+^ T cells versus naive CD8^+^ T cells from their SHP-1-sufficient IL1r1^−/−^ littermates (SHP-1^WT^). SHP-1^null^ CD8^+^ T cells maintained a superior ability to prevent tumor nodule formation compared with their SHP-1^WT^ counterparts (Figure 1d), similar to the results using IL1r1-sufficient cells.

**Figure 2:**
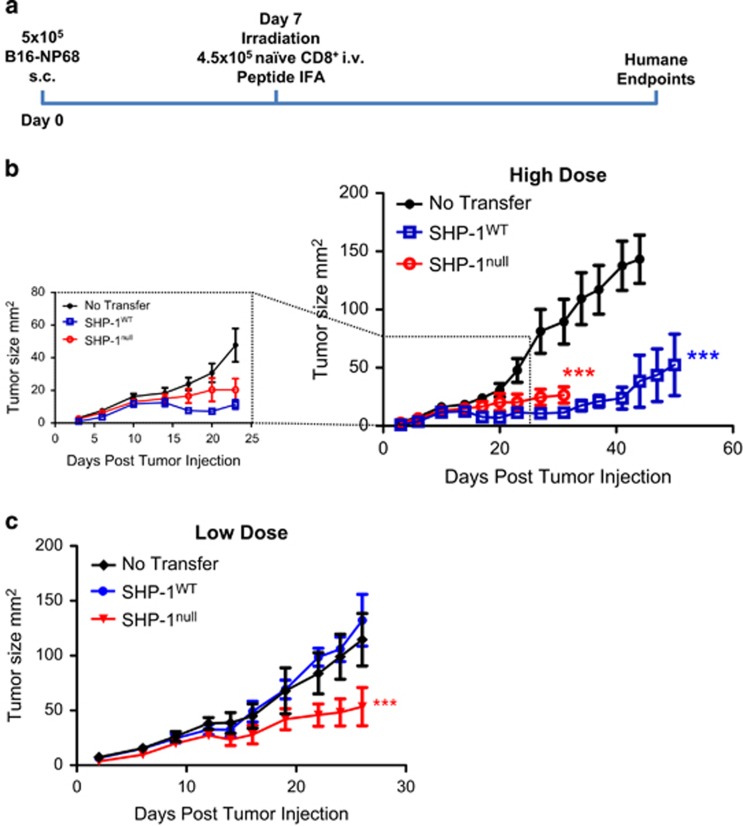
SHP-1^null^ cells only offer improved tumor control at low cell doses. (**a**) Schematic representation of therapeutic solid tumor model. (**b**) Tumor growth curves for C57BL/6 hosts treated with 4.5 × 10^5^ SHP-1^null^ or SHP-1^WT^ CD8^+^ T cells (*n*=9–11). Expanded section shows early stage growth, up to day 23, in more detail. Differences in tumor growth rate were calculated by linear regression. ^***^P⩽0.001. (**c**) Tumor growth curves for tumor-bearing hosts treated with 5 × 10^4^ SHP-1^null^ or SHP-1^WT^ CD8^+^ T cells (*n*=6–8). Differences in tumor growth rate were calculated by linear regression. ^***^P⩽0.001.

To demonstrate the therapeutic potential of SHP-1^null^ CD8^+^ T cells, NP68-B16 cells were introduced subcutaneously into host mice in a solid tumor model (Figure 2a). Tumors were established for 7 days and then treated with 4.5 × 10^5^ magnetically sorted CD8^+^ T cells. Both SHP-1^WT^ CD8^+^ T cells and SHP-1^null^ CD8^+^ T cells significantly controlled tumor growth (Figure 2b), however, SHP-1^WT^ cells were more effective. At day 17, SHP-1^WT^ CD8^+^ T cells caused tumor regression in all mice. In contrast, no regression was observed among mice treated with SHP-1^null^ CD8^+^ T cells. This observation suggests that the absence of SHP-1 offers no benefit in the treatment of tumors at this T cell dose (high dose) of cells. When the cell dose was reduced to 5 × 10^4^ CD8^+^ T cells per mouse (low dose), only SHP-1^null^ cells were able control tumor growth (Figure 2c).

### A unique SHP-1^null^ population transplants me/me pathology to tumor-bearing hosts

**Figure 3:**
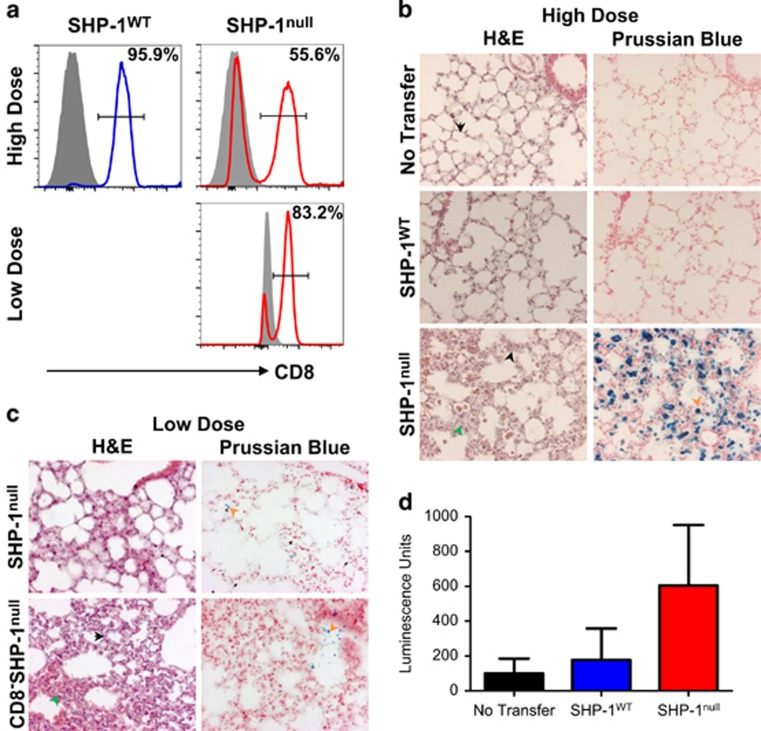
Contaminating CD8^−^ cells from SHP-1^null^ donors causes fatal lung pathology. (**a**) Flow cytometric analysis of CD8-stained, magnetically enriched donor cells used in ‘high dose' (Figure 2b) or ‘low dose' (Figure 2c). Gray peak represents fluorescence minus CD8 antibody. (**b**) Lungs from hosts receiving high cell doses. Sections were stained with H&E or Prussian Blue. Images were acquired at × 20 magnification. (**c**) Lungs from hosts receiving low cell doses. Sections were stained with H&E or Prussian Blue. Black arrows indicate alveolar septa, green arrows indicate fibrosis, orange arrows indicate hemosiderin. Images were acquired at × 20 magnification. (**d**) Luciferase expression measured in lung lysates from hosts receiving high cell doses.

There was a marked weight loss and declining health among the animals treated with high-dose SHP-1^null^ cells, necessitating euthanasia by day 31. Post-mortem analysis revealed considerable lung pathology, not seen in the lung tumor model, but known to be a feature of the motheaten mouse. Analysis of the donor cells revealed that despite magnetic enrichment of CD8^+^ cells before transfer, a large population of CD8^−^ live cells persisted in the high-dose SHP-1^null^ donor cells, and to a lesser extent in the low-dose SHP-1^null^, which was not present in the SHP-1^WT^ donor cells (Figure 3a).

Histological analysis of the lungs revealed marked fibrosis, leukocyte infiltration and evidence of hemorrhage (Prussian Blue staining indicates hemosiderin, generally macrophage-associated) in the lungs of mice receiving high-dose SHP-1^null^ cells (Figure 3b). Luciferase was also detected in the lungs of high-dose SHP-1^null^ cell recipients, suggesting the presence of NP68-B16 tumor cells (Figure 3d). In contrast, mice receiving either no T cells or SHP-1^WT^ cells had histologically healthy lungs, excluding a role for radiation in the observed pathology. Mice receiving low-dose SHP-1^null^ cells with a lower proportion of contaminating cells exhibited less pathology (Figure 3c, upper panel). Moreover, transfer of a low dose of the contaminating CD8^−^ cells alone, isolated by flow sorting of magnetically enriched cells (Figure 3c, lower panel), caused fibrosis and leukocyte infiltration comparable to the high-dose SHP-1^null^ cells, indicating that it is not the cell dose, but the presence of this contaminating population that is pathological. These findings concur with the current understanding of me/me lung pathology being CD8^+^ T-cell-independent.^19, 20^

Detailed analysis of the magnetically enriched cell population by flow cytometry indicated the presence of B cells (CD19^+^), natural killer cells (NK1.1^+^), and both lymphoid and myeloid dendritic cells (CD11c^+^ and either CD8^+^ or CD11b^+^, respectively) in the transferred SHP-1^null^ cells (Supplementary Fig. 1). Smaller populations of monocytes and macrophages were also detected (CD11b^+^, Ly6G^−^, Ly6C^+/−^). Any of these contaminating subsets, either alone or in combination, could be responsible for the observed pathology.^17, 18, 19^ It remains unclear how such diverse cell populations escaped capture by isolation kits incorporating antibodies specific for a number of markers detected in subsequent flow cytometry experiments, including CD11b, CD11c, CD19 and MHC-class II. However, this phenomenon appears linked in some way to the absence of SHP-1 in hematopoietic cell lineages.

### Purified CD8^+^ SHP-1^null^ T cells control tumors without pathology

**Figure 4:**
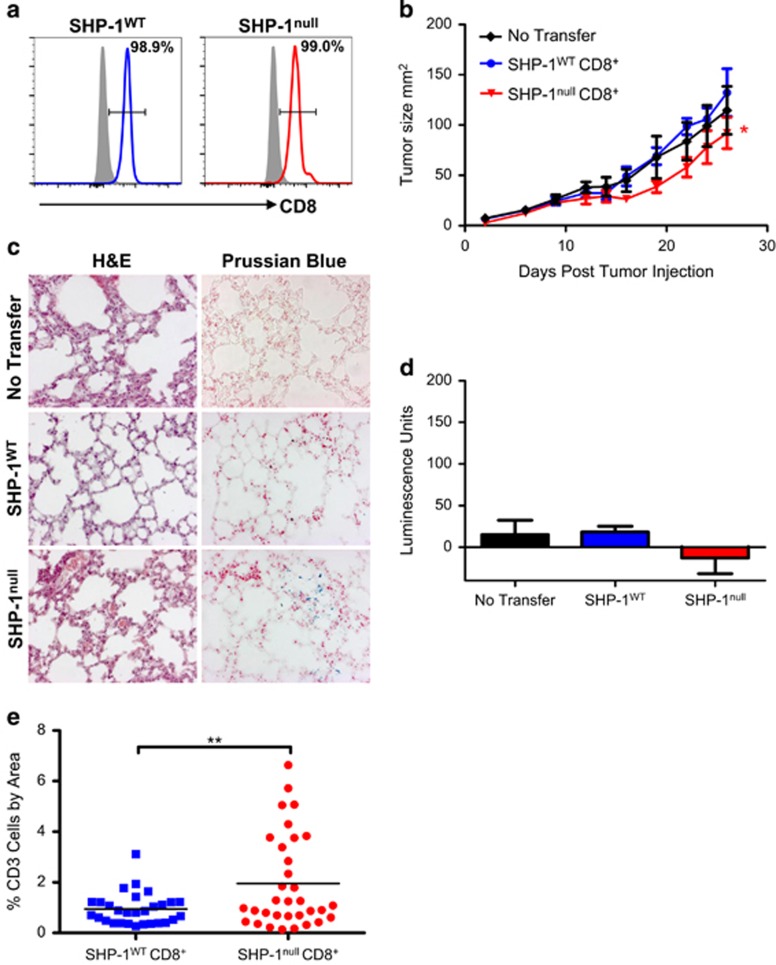
Flow-sorted SHP-1^null^ naive CD8^+^ T cells control tumor growth without lung pathology. (**a**) Flow cytometric analysis of magnetically enriched and flow-sorted donor cells. (**b**) Tumor growth curves for hosts treated with low dose, enriched/sorted SHP-1^null^ or SHP-1^WT^ CD8^+^ T cells (*n*=8–9). ‘No transfer' and ‘SHP-1^WT^' are the same groups shown in Figure 2c, conducted at the same time. Differences in tumor growth rate were calculated by linear regression. *P⩽0.05. (**c**) H&E and Prussian Blue staining of lungs from tumor-bearing hosts shown in **b**. (**d**) Luciferase expression measured in lung lysates from hosts shown in **b**. (**e**) Quantification of CD3 staining in tumor sections taken from Figure 4c. One dot=one field of view (5 fields of view per tumor). Significance was calculated using a two-tailed unpaired Student's *t-*test. ^**^P⩽0.01.

To eliminate contamination, magnetically enriched CD8^+^ cells were further sorted by flow cytometry. Naive CD8^+^ T cells were >98% pure (Figure 4a). These cells were transferred at low dose to tumor-bearing hosts 7 days after subcutaneous injection of tumor cells. The purified SHP-1^null^ CD8^+^ cells still offered improved control of tumor growth (Figure 4b), although more modestly than the less-pure cell population (Figure 2c), indicating that the contaminating cells were anti-tumoral as well as pathological. Me/me-like lung pathology was not observed in any of the hosts (Figure 4c) and luminescent tumor cells were not detected in the lungs (Figure 4d).

These data show that relief of SHP-1-mediated T-cell inhibition is a feasible strategy to improve the success rates of adoptive T-cell transfer therapy, supporting previous investigations using a disseminated leukemia model.^9^ Our earlier work indicated that one mechanism by which SHP-1 deletion suppresses tumor growth is through increased expansion of effector T cells.^7, 21^ In the present study, we observed a >15-fold expansion of SHP-1^null^ T cells relative to SHP-1^WT^ T cells in tumor-draining lymph nodes 7 days after transfer into a tumor-bearing host (data not shown). Furthermore, histological analysis of tumor tissue recovered from the experiment shown in Figure 4b revealed significantly increased numbers of T cells in those tumors that were treated with SHP-1^null^ T cells (Figure 4e). The cell dose-dependent action of SHP-1 inhibition in this system also suggests that increased proliferation is important, and is an appealing aspect of this strategy given that tumor-specific T cells are a limited resource in the clinic. If this strategy was translated to the clinic, contamination by other SHP-1^null^ lineages would be unlikely in adoptive transfer strategies, as only CD8^+^ T cells would be manipulated for transfer. However, the pathology associated with transfer of non-CD8^+^ SHP-1^null^ lineages revealed in this study is an important consideration for systemic inhibitor-based strategies, which would result in SHP-1 inhibition across multiple cell types.

It should be noted that the anti-viral F5 TCR operates at a higher affinity than tumor-specific TCRs.^22^ The effect of SHP-1 inhibition may therefore be more pronounced in the clinic due to a greater impact on activation thresholds at suboptimal TCR affinities. Furthermore, the B16 melanoma line is poorly immunogenic and cannot be controlled with anti-CTLA-4 therapy alone.^23^ These considerations further suggest that the modest control of B16 growth demonstrated here may translate to more profound effects in humans.

The question of how other checkpoint inhibitors might interact with SHP-1 inhibition strategies is an important one. A recent study demonstrated a broad role for SHP-1 in the regulation of CD8^+^ T cells with a range of TCR affinities, whereas PD-1 is preferentially expressed alongside higher affinity TCRs.^24^ In addition, the interaction between SHP-1 and CTLA-4 is limited.^25^ These findings suggest that combining SHP-1 inhibition with PD-1 blockade would be more likely to result in synergy than redundancy. SHP-1 modulates the activity of several proteins that become activated following TCR engagement, including CD3ζ, lymphocyte-specific protein tyrosine kinase (Lck), ζ-chain-associated protein kinase 70 (ZAP70), phosphoinositide 3 kinase (PI3K) and the ‘onc F' proto-oncogene (Vav1), and is known to bind the inhibitory leukocyte-associated immunoglobulin-like receptor-1 (LAIR-1).^21, 26, 27, 28, 29, 30, 31^ The genetic model used here supports the development of translational approaches, such as genome editing using CRISPR or TALENs,^32, 33, 34, 35^ to manipulate SHP-1 expression in human T cells to augment cancer immunotherapy.

## Methods

### Mice

Motheaten heterozygous (C57BL/6J me^+/−^) mice were obtained from the Jackson Laboratory, and the transgenic F5 TCR was introduced as described previously.^15^ F5^+/+^ me^+/−^ mice were then crossed with IL1r1^−/−^ mice (B6.126S7-IL1r1^tm1Imx^/J). Genotyping was performed as described previously for F5 and me,^15^ and following standard JAX protocols for IL1r1 (http://jaxmice.jax.org/strain/003245.html). Mice were bred to homozygosity for F5 and IL1r1 knockout, and F5^+/+^IL1r1^−/−^me^+/−^ were bred to produce F5^+/+^IL1r1^−/−^me^−/−^ offspring (SHP-1^null^). CD8^+^ cells were isolated from these mice and their WT littermates (SHP-1^WT^, confirmed by PCR) at 8–12 weeks of age. Eight-to-nine-week-old C57BL/6J tumor host mice were purchased from Charles River. Hosts and T-cell donors were gender-matched. Mice had free access to food and water, and were housed and maintained according to Home Office standards and the Animals (Scientific Procedures) Act 1986.

### Adoptive cell transfer

CD8^+^ T cells were enriched from splenocytes by negative selection using a CD8a^+^ T-cell isolation kit with LS columns, according to the manufacturer's instructions (Miltenyi Biotec Ltd, Bisley, Surrey, UK). Isolated cells were frozen until required. Thawed cells were recovered at 37 °C for a minimum of 4 h, and either used directly or further purified by flow sorting, as indicated. Flow-sorted cells were stained with anti-TCR-FITC, anti-CD4-PE, anti-CD8-APC, anti-CD11c-PE, anti-CD19PECy7, anti-CD27-BV421 and anti-CD44-APCCy7 (BioLegend, London, UK). Dead cells were excluded using Zombie Aqua (eBioscience Ltd, Hatfield, UK). Cells were sorted using a custom-modified FACSAria II flow cytometer (BD Biosciences, 9320 Erembodegem, Belgium). Voltages were set using OneComp eBeads (eBioscience) and Arc-reactive beads (Invitrogen, Life Technologies Ltd, Paisley, UK). The flow-sorted naive CD8^+^ fraction was defined as live single cells with the following phenotype: TCR^+^, CD4^−^, CD8^+^, CD11c^−^, CD19^−^, CD27^+^, CD44^−^. Cells that were positive for CD4, CD11c and CD19 were sorted into the CD8^−^ fraction. CD8^+^ cells that were positive for CD44 or negative/dim for CD27 were discarded. Cells were counted using a hemocytometer and resuspended in PBS for injection.

### Flow cytometry

Cells were stained with LIVE/DEAD Fixable Aqua (Invitrogen), treated with 50 nM dasatinib (Sigma-Aldrich Co Ltd, Dorset, UK) to prevent TCR downregulation, then stained with PE-conjugated NP68 tetramer at 37 °C, followed by surface antibodies at 4 °C as follows: anti-TCRVβ11-FITC, anti-CD8-PerCPCy5.5, anti-CD27-BV421, anti-CD44-APCCy7, anti-CD62L-PECy7 and anti-CD69-APC (BioLegend). Cells were fixed and analyzed using a FACSCanto II flow cytometer (BD Biosciences). Voltages were set using OneComp eBeads (eBioscience) and Arc-reactive beads (Invitrogen). CytoCount beads were used to quantify cell numbers, according to the manufacturer's instructions (Dako UK Ltd, Ely, UK).

### Tumor cell line

The murine melanoma cell line B16F10 (ATCC number CRL-6475) was transduced via retroviral infection in the presence of 10 μg ml^−1^ protamine sulfate (Sigma) to express luciferase and a YFP reporter (separated by an IRES). Two rounds of infection were performed, and YFP^+^ cells were identified using a FACSCalibur flow cytometer (BD Biosciences). YFP^+^ cells were cloned and assayed for luciferase activity. A monoclonal luciferase^+^ line was then super-infected with a retrovirus encoding the class I-restricted influenza nucleoprotein epitope NP68 as a fusion protein with GFP.^36^ These cells were sorted by flow cytometry as described above and cloned. All experiments were performed using a single mycoplasma-free line of monoclonal luciferase^+^ NP68^+^ B16F10 cells, designated NP68-B16.

### Luciferase quantification in lungs

The left lung node was collected in PBS, and homogenized to a single-cell suspension. Cells were lysed with passive lysis buffer (Promega UK, Southampton, UK), and the lysate was stored at −20 °C until required. All lysates for a single experiment were assayed together in triplicate. Luciferase activity was quantified *in vitro* using a Dual-Luciferase Reporter Assay (Promega) and measured using a FLUOstar OPTIMA (BMG Labtech Ltd, Aylesbury, UK).

### Tumor metastasis model

C57BL/6J mice were exposed to 650 cGy total body irradiation (TBI) on day 1. On day 2, 3.4 × 10^4^ naive T cells were transferred intravenously, followed by subcutaneous administration of 100 μg NP68 peptide (ASNENMDAM, Peptide Synthetics) in incomplete Freund's adjuvant (peptide IFA, final volume of 200 μl). The next day, 2.5 × 10^6^ NP68-B16 tumor cells were transferred intravenously. Mice were monitored until day 13 or 14 and then killed. The lungs were excised and fixed in 4% formalin. Tumor metastasis nodules were enumerated macroscopically up to a maximum detection limit of 425 nodules.

### Solid tumor model

NP68-B16 cells (5 × 10^5^) were injected subcutaneously into the shaven left flank. Tumors were measured with callipers, and tumor size was calculated as the product of two perpendicular diameters. On day 7, tumor-bearing hosts were irradiated with 650 cGy TBI and then treated with peptide IFA and naive T cells, at either a high dose of 4.5 × 10^5^ cells per mouse or a low dose of 5 × 10^4^ cells per mouse, unless stated otherwise.

### Immunohistochemistry

Formalin-fixed paraffin-embedded lung tissue was cut into 5-μm sections and stained with either hematoxylin and eosin (H&E, Sigma) or Prussian Blue using standard histological techniques. Prussian Blue staining was performed after rehydration using freshly prepared 2% potassium ferrocyanide (Sigma) and 2% hydrochloric acid (Fisher Chemical, Loughborough, UK). One hour later, sections were rinsed several times in distilled water, counterstained with neutral red (Sigma), dehydrated and mounted with DPX (Fisher Chemical). Images were taken using an Axio microscope with AxioVision software (Zeiss). Formalin-fixed paraffin-embedded tumor tissue was cut into 5-μm sections and rehydrated. Antigen retrieval was performed using Tris-EDTA buffer (10 mM Tris, 1 mM EDTA, pH 9). Sections were blocked with 0.5% H_2_O_2_ in methanol, followed by goat serum (ImmPRESS Reagent Kit, Vector Laboratories, Peterborough, UK). After staining with rat anti-CD31 (DIA-310, Dianova) and goat anti-rat linked to horseradish peroxidise (HRP; Vector Laboratories Ltd), sections were developed for 30 s with 3,3′-diaminobenzidine (DAB). Slides were then rinsed in PBS, blocked with horse serum (Vector Laboratories) and stained overnight with rabbit anti-CD3 (A0452, Dako). After staining with horse anti-rabbit-HRP (Vector Laboratories), sections were developed for 90–120 s with SG detection solution (Vector Laboratories). Slides were mounted using DPX (Fisher Chemical). Sections were photographed at × 20 magnification using an EVOS XL Core Microscope (Thermo Fisher Scientific, Peterborough, UK).

### Quantification of CD3 staining

The whole tumor area was photographed at × 20 magnification. Five sequentially numbered fields of view (FOV) were analyzed per tumor. Where 5–6 FOV were taken, images 1–5 were analyzed; for 7–8 FOV, images 1, 3–5, 7 were analyzed; for 9 FOV, images 1, 3, 5, 7 and 9 were analyzed. Images were analyzed using the Fiji version of ImageJ. The scale was set to 3.4 pixels=1 μm (based on image scale bar). Non-specific staining was subtracted using Background Correction, and the images were separated into FastRed, FastBlue and DAB using Color Deconvolution. The FastBlue image was used to analyze CD3. Minimum image threshold was set at 87, and maximum image threshold was set at 173–210. Percent area was analyzed and plotted. In cases where the tumor section did not cover the whole FOV, the blank area was subtracted before analysis. Six tumors were analyzed per treatment group.

### Statistics and figures

Statistical analyses were performed in Prism 5 (GraphPad Software Inc, La Jolla, CA, USA). Figures were prepared using FlowJo software (TreeStar Inc, Ashland, OR, USA), Powerpoint (Microsoft, Reading, UK) and Prism 5 (GraphPad Software Inc.). Mean values are presented with s.e.m.

## References

[bib1] Topalian SL, Hodi FS, Brahmer JR, Gettinger SN, Smith DC, McDermott DF et al. Safety, activity, and immune correlates of anti–PD-1 antibody in cancer. N Engl J Med 2012; 366: 2443–2454. 22658127 10.1056/NEJMoa1200690 PMC3544539

[bib2] Hodi FS, O'Day SJ, McDermott DF, Weber RW, Sosman JA, Haanen JB et al. Improved survival with ipilimumab in patients with metastatic melanoma. N Engl J Med 2010; 363: 711–723. 20525992 10.1056/NEJMoa1003466 PMC3549297

[bib3] Wolchok JD, Kluger H, Callahan MK, Postow MA, Rizvi NA, Lesokhin AM et al. Nivolumab plus ipilimumab in advanced melanoma. N Engl J Med 2013; 369: 122–133. 23724867 10.1056/NEJMoa1302369 PMC5698004

[bib4] Lorenz U. SHP-1 and SHP-2 in T cells: two phosphatases functioning at many levels. Immunol Rev 2009; 228: 342–359. 19290938 10.1111/j.1600-065X.2008.00760.x PMC2669678

[bib5] Matthews RJ, Bowne DB, Flores E, Thomas ML. Characterization of hematopoietic intracellular protein tyrosine phosphatases: description of a phosphatase containing an SH2 domain and another enriched in proline-, glutamic acid-, serine-, and threonine-rich sequences. Mol Cell Biol 1992; 12: 2396–2405. 1373816 10.1128/mcb.12.5.2396-2405.1992 PMC364412

[bib6] Carter JD, Neel BG, Lorenz U. The tyrosine phosphatase SHP-1 influences thymocyte selection by setting TCR signaling thresholds. Int Immunol 1999; 11: 1999–2014. 10590266 10.1093/intimm/11.12.1999

[bib7] Sathish JG, Dolton G, LeRoy FG, Matthews RJ. Loss of Src homology region 2 domain-containing protein tyrosine phosphatase-1 increases CD8+ T cell-APC conjugate formation and is associated with enhanced *in vivo* CTL function. J Immunol 2007; 178: 330–337. 17182570 10.4049/jimmunol.178.1.330

[bib8] Tai W-T, Cheng A-L, Shiau C-W, Liu C-Y, Ko C-H, Lin M-W et al. Dovitinib induces apoptosis and overcomes sorafenib resistance in hepatocellular carcinoma through SHP-1–mediated inhibition of STAT3. Mol Cancer Ther 2012; 11: 452–463. 22180308 10.1158/1535-7163.MCT-11-0412

[bib9] Stromnes IM, Fowler C, Casamina CC, Georgopolos CM, McAfee MS, Schmitt TM et al. Abrogation of Src homology region 2 domain-containing phosphatase 1 in tumor-specific T cells improves efficacy of adoptive immunotherapy by enhancing the effector function and accumulation of short-lived effector T cells *in vivo*. J Immunol 2012; 189: 1812–1825. 22798667 10.4049/jimmunol.1200552 PMC3522079

[bib10] Naing A, Reuben JM, Camacho LH, Gao H, Lee BN, Cohen EN et al. Phase I dose escalation study of sodium stibogluconate (SSG), a protein tyrosine phosphatase inhibitor, combined with interferon alpha for patients with solid tumors. J Cancer 2011; 2: 81–89. 21326629 10.7150/jca.2.81 PMC3039225

[bib11] Chen L, Sung S-S, Yip MLR, Lawrence HR, Ren Y, Guida WC et al. Discovery of a novel Shp2 protein tyrosine phosphatase inhibitor. Mol Pharmacol 2006; 70: 562–570. 16717135 10.1124/mol.106.025536

[bib12] Kundu S, Fan K, Cao M, Lindner DJ, Zhao ZJ, Borden E et al. Novel SHP-1 inhibitors tyrosine phosphatase inhibitor-1 and analogs with preclinical anti-tumor activities as tolerated oral agents. J Immunol 2010; 184: 6529–6536. 20421638 10.4049/jimmunol.0903562 PMC3049920

[bib13] Yi T, Pathak MK, Lindner DJ, Ketterer ME, Farver C, Borden EC. Anticancer activity of sodium stibogluconate in synergy with IFNs. J Immunol 2002; 169: 5978–5985. 12421984 10.4049/jimmunol.169.10.5978

[bib14] Zhang Y-L, Keng Y-F, Zhao Y, Wu L, Zhang Z-Y. Suramin is an active site-directed, reversible, and tight-binding inhibitor of protein-tyrosine phosphatases. J Biol Chem 1998; 273: 12281–12287. 9575179 10.1074/jbc.273.20.12281

[bib15] Johnson KG, LeRoy FG, Borysiewicz LK, Matthews RJ. TCR signaling thresholds regulating T cell development and activation are dependent upon SHP-1. J Immunol 1999; 162: 3802–3813. 10201897

[bib16] Shultz LD. Pleiotropic effects of deleterious alleles at the "motheaten" locus. Curr Top Microbiol Immunol 1988; 137: 216–222. 3416633 10.1007/978-3-642-50059-6_32

[bib17] Croker BA, Lawson BR, Rutschmann S, Berger M, Eidenschenk C, Blasius AL et al. Inflammation and autoimmunity caused by a SHP1 mutation depend on IL-1, MyD88, and a microbial trigger. Proc Natl Acad Sci USA 2008; 105: 15028–15033. 18806225 10.1073/pnas.0806619105 PMC2567487

[bib18] Shultz LD, Coman DR, Bailey CL, Beamer WG, Sidman CL. "Viable motheaten," a new allele at the motheaten locus. I. Pathology. Am J Pathol 1984; 116: 179–192. 6380298 PMC1900532

[bib19] Abram CL, Roberge GL, Pao LI, Neel BG, Lowell CA. Distinct roles for neutrophils and dendritic cells in inflammation and autoimmunity in motheaten mice. Immunity 2013; 38: 489–501. 23521885 10.1016/j.immuni.2013.02.018 PMC3613338

[bib20] Kaneko T, Saito Y, Kotani T, Okazawa H, Iwamura H, Sato-Hashimoto M et al. Dendritic cell-specific ablation of the protein tyrosine phosphatase Shp1 promotes Th1 cell differentiation and induces autoimmunity. J Immunol 2012; 188: 5397–5407. 22539788 10.4049/jimmunol.1103210

[bib21] Sathish JG, Johnson KG, LeRoy FG, Fuller KJ, Hallett MB, Brennan P et al. Requirement for CD28 co-stimulation is lower in SHP-1-deficient T cells. Eur J Immunol 2001; 31: 3649–3658. 11745385 10.1002/1521-4141(200112)31:12<3649::aid-immu3649>3.0.co;2-8

[bib22] Aleksic M, Liddy N, Molloy PE, Pumphrey N, Vuidepot A, Chang K-M et al. Different affinity windows for virus and cancer-specific T-cell receptors – implications for therapeutic strategies. Eur J Immunol 2012; 42: 3174–3179. 22949370 10.1002/eji.201242606 PMC3776049

[bib23] van Elsas A, Hurwitz AA, Allison JP. Combination immunotherapy of B16 melanoma using anti-cytotoxic T lymphocyte-associated antigen 4 (CTLA-4) and granulocyte/macrophage colony-stimulating factor (GM-CSF)-producing vaccines induces rejection of subcutaneous and metastatic tumors accompanied by autoimmune depigmentation. J Exp Med 1999; 190: 355–366. 10430624 10.1084/jem.190.3.355 PMC2195583

[bib24] Hebeisen M, Baitsch L, Presotto D, Baumgaertner P, Romero P, Michielin O et al. SHP-1 phosphatase activity counteracts increased T cell receptor affinity. J Clin Invest 2013; 123: 1044–1056. 23391724 10.1172/JCI65325 PMC3582132

[bib25] Zhang Y, Allison JP. Interaction of CTLA4 with AP50, a clathrin-coated pit adaptor protein. Proc Natl Acad Sci USA 1997; 94: 9273–9278. 9256472 10.1073/pnas.94.17.9273 PMC23153

[bib26] Watson HA, Wehenkel S, Matthews RJ, Ager A. SHP-1: the next checkpoint target for cancer immunotherapy? Biochem Soc Trans 2016; 44: 356–362. 27068940 10.1042/BST20150251 PMC5264497

[bib27] Chiang GG, Sefton BM. Specific dephosphorylation of the Lck tyrosine protein kinase at Tyr-394 by the SHP-1 protein-tyrosine phosphatase. J Biol Chem 2001; 276: 23173–23178. 11294838 10.1074/jbc.M101219200

[bib28] Cuevas B, Lu Y, Watt S, Kumar R, Zhang J, Siminovitch KA et al. SHP-1 regulates Lck-induced phosphatidylinositol 3-kinase phosphorylation and activity. J Biol Chem 1999; 274: 27583–27589. 10488096 10.1074/jbc.274.39.27583

[bib29] Plas DR, Johnson R, Pingel JT, Matthews RJ, Dalton M, Roy G et al. Direct regulation of ZAP-70 by SHP-1 in T cell antigen receptor signaling. Science 1996; 272: 1173–1176. 8638162 10.1126/science.272.5265.1173

[bib30] Sozio MS, Mathis MA, Young JA, Walchli S, Pitcher LA, Wrage PC et al. PTPH1 is a predominant protein-tyrosine phosphatase capable of interacting with and dephosphorylating the T cell receptor zeta subunit. J Biol Chem 2004; 279: 7760–7769. 14672952 10.1074/jbc.M309994200

[bib31] Stebbins CC, Watzl C, Billadeau DD, Leibson PJ, Burshtyn DN, Long EO. Vav1 dephosphorylation by the tyrosine phosphatase SHP-1 as a mechanism for inhibition of cellular cytotoxicity. Mol Cell Biol 2003; 23: 6291–6299. 12917349 10.1128/MCB.23.17.6291-6299.2003 PMC180957

[bib32] Poirot L, Philip B, Schiffer-Mannioui C, Le Clerre D, Chion-Sotinel I, Derniame S et al. Multiplex genome-edited T-cell manufacturing platform for “off-the-shelf” adoptive T-cell immunotherapies. Cancer Res 2015; 75: 3853–3864. 26183927 10.1158/0008-5472.CAN-14-3321

[bib33] Schumann K, Lin S, Boyer E, Simeonov DR, Subramaniam M, Gate RE et al. Generation of knock-in primary human T cells using Cas9 ribonucleoproteins. Proc Natl Acad Sci USA 2015; 112: 10437–10442. 26216948 10.1073/pnas.1512503112 PMC4547290

[bib34] Chen YY. Efficient gene editing in primary human T cells. Trends Immunol 2015; 36: 667–669. 26440702 10.1016/j.it.2015.09.001

[bib35] Lloyd A, Vickery ON, Laugel B. Beyond the antigen receptor: editing the genome of T-cells for cancer adoptive cellular therapies. Front Immunol 2013; 4: 221. 23935598 10.3389/fimmu.2013.00221 PMC3733021

[bib36] Cuff S, Dolton G, Matthews RJ, Gallimore A. Antigen specificity determines the pro- or antitumoral nature of CD8+ T cells. J Immunol 2010; 184: 607–614. 20007540 10.4049/jimmunol.0804089

