# Supplementary material for: Purity of transferred CD8+ T cells is crucial for safety and efficacy of combinatorial tumor immunotherapy in the absence of SHP-1
Source: Immunol Cell Biol. 2016 Jul 19;94(8):802–8. doi: 10.1038/icb.2016.45 (PMC5027373; doi:10.1038/icb.2016.45)
Supplement: Supplementary information [file icb201645x1.pdf]

SUPPLEMENTARY INFORMATION

Detailed flow cytometric analysis of the contaminating cell population is presented here.

METHODS

Lymphocytes isolated from the spleens and peripheral lymph nodes of SHP-1^{WT} or SHP-1^{null} mice were thawed and recovered for 1 hr at 37°C. CD8^{+} cells were magnetically-enriched according to the manufacturer's instructions (Miltenyi Biotec). Cells were stained with anti-CD3-PECy5 (eBioscience), anti-CD4-BrilliantViolet785, anti-CD8-BrilliantViolet711, anti-CD11b-AlexaFluor488, anti-CD11c-APC, anti-CD19-PECy7, anti-Ly6C-PE, anti-Ly6G-PEDazzle594, anti-MHC class II-APCCy7, anti-NK1.1-BrilliantViolet421 (all Biolegend), and LIVE/DEAD Fixable Aqua (Invitrogen). Data were acquired using a custom-modified FACSAria II flow cytometer (BD Biosciences) and analysed with FlowJo software (TreeStar Inc.). Gates were set using fluorescence minus one controls (not shown). The plots shown were pre-gated to identify live single lymphocytes on the basis of light scatter.

FIGURE LEGEND

Supplementary Figure 1: Analysis of magnetically-enriched cells by flow cytometry. (**A**) SHP-1^{WT} cells were stained for the indicated markers and analysed by flow cytometry. Numbers indicate percentage of immediate parent population. (**B**) SHP-1^{null} cells were stained and analysed in the same way.

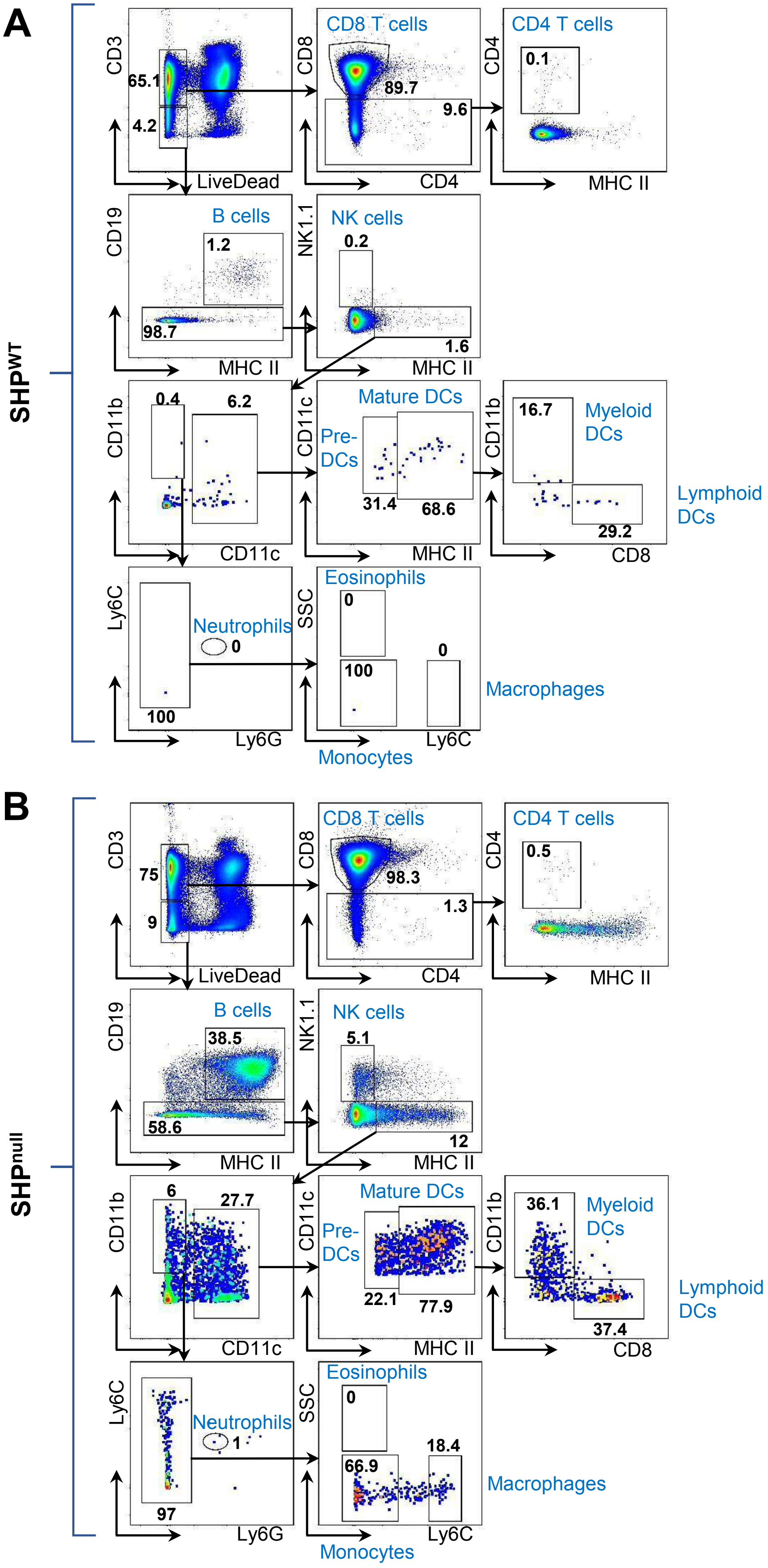

A
SHPWT
CD3
LiveDead
65.1
4.2
CD8 T cells
CD8
CD4
89.7
9.6
CD4 T cells
0.1
MHC II
B cells
CD19
1.2
98.7
NK cells
NK1.1
0.2
1.6
CD11b
CD11c
0.4
6.2
Mature DCs
Pre-DCs
31.4
68.6
16.7
Myeloid DCs
Lymphoid DCs
29.2
CD8
Ly6C
Ly6G
Neutrophils
0
100
Eosinophils
SSC
0
100
0
Macrophages
Monocytes
B
SHPnull
75
9
98.3
1.3
0.5
38.5
58.6
5.1
12
6
27.7
22.1
77.9
36.1
37.4
1
97
66.9
18.4
